# Addition of Selenium to Silver Nanoparticles: An Approach to Enhance Their Antimicrobial and Biological Properties – Systematic Review

**DOI:** 10.1007/s12011-026-05184-5

**Published:** 2026-06-25

**Authors:** João Marcos Carvalho-Silva, Andréa Cândido dos Reis

**Affiliations:** Department of Dental Materials and Prosthesis, Ribeirão Preto School of Dentistry, University of São Paulo (USP), Avenida Do Café, s/n, Ribeirão Preto, São Paulo 14040-904 Brazil

**Keywords:** Silver, Selenium, Nanoparticles, Antimicrobial, Cytotoxicity, Nanomedicine

## Abstract

The aim was to address the question, “Can the addition of selenium nanoparticles (SeNPs) to silver nanoparticles (AgNPs) enhance their antimicrobial and biological properties?” This systematic review followed the Preferred Reporting Items for Systematic Reviews and Meta-Analyses (PRISMA) 2020 guidelines and was registered in the Open Science Framework (OSF). A comprehensive search was conducted in Scopus, PubMed, Embase, Lilacs, ScienceDirect, and the gray literature databases (Google Scholar and ProQuest) to identify in vitro studies evaluating the effect of SeNPs incorporation into AgNPs (Se-AgNPs) on antimicrobial activity and biological behavior. Se-AgNPs demonstrated enhanced antimicrobial and antibiofilm activity against both Gram-positive and Gram-negative bacteria, as well as fungal strains. Additionally, they exhibited improved cell adhesion, viability, and proliferation, along with reduced cytotoxicity. Se-AgNPs showed a synergistic enhancement of antimicrobial and antibiofilm activity, with a potential reduction in cytotoxic effects. Nevertheless, the small number of studies limits the strength of the conclusions, underscoring the need for rigorous, standardized investigations to strengthen or refute these findings.

**Supplementary Information:**

The online version contains supplementary material available at 10.1007/s12011-026-05184-5.

## Introduction

Interdisciplinary advances in biotechnology, chemistry, physics, and engineering have enabled the rational design and functionalization of nanomaterials to optimize their biological and antimicrobial performance [1–13]. Representative examples include metallic nanoparticles (e.g., silver [AgNPs] and gold) [14–16], trace elements nanoparticles (selenium [SeNPs]) [17, 18], organic nanoparticles (e.g., chitosan, liposomes, and polylactic acid) [19–21], and carbon-based nanomaterials (e.g., graphene, reduced graphene oxide, and carbon nanotubes) [22–24].

Among these, the combination of AgNPs and SeNPs (Se-AgNPs) represents a novel approach that has not yet been systematically reviewed. Such an analysis may clarify how these hybrid nanoparticles (NPs) exert their antimicrobial effects. AgNPs are widely recognized for their potent antimicrobial and antibiofilm properties, low cytotoxicity at low concentrations, and anti-inflammatory potential [1–3, 14, 15]. Functionalization with SeNPs provides additional benefits, including enhanced antimicrobial efficacy, antioxidant and immunomodulatory effects, and a reduction in the toxicity typically associated with AgNPs [4–13].

AgNPs have demonstrated significant potential in combating drug-resistant biofilms, including those formed by *Candida* spp [1, 2, 15, 25–27]., *Staphylococcus aureus* (including methicillin-resistant [MRSA] and vancomycin-resistant [VRSA] strains) [28–30], and *Pseudomonas aeruginosa* [28–30], which are responsible for systemic infections with high mortality rates (approximately 60%). Their efficacy is largely attributed to their ability to penetrate and disrupt the extracellular polysaccharide matrix (EPS) [28–30]. However, challenges remain, including limited colloidal stability, high rates of ion release, and dose-dependent toxicity, which may induce oxidative stress in healthy cells [31–33].

The combination of AgNPs with antioxidant and cytoprotective agents such as SeNPs may reduce the required concentration of silver ions (Ag⁺), broaden the antimicrobial spectrum, and enhance chemical stability under variations in temperature and pH [9, 11, 34–41]. Although isolated SeNPs exhibit limited antimicrobial activity due to their low reactive oxygen species (ROS) generation and the formation of complexes that inhibit microbial metabolism [9, 11], they are notable for their strong cytoprotective effects [35, 42–45]. These include modulation of oxidative stress via selenoproteins including glutathione peroxidase (GSH-Px) and thioredoxin reductase (TrxR), as well as the prevention of apoptosis through specific cellular pathways [35, 42–45]. In addition, SeNPs interact with free Ag^+^, promoting their elimination as selenides and thereby reducing the **risk** of bioaccumulation and toxicity [11, 35, 42, 43, 46].

AgNPs have previously been modified to improve their applicability and antimicrobial efficacy through incorporation into metal-organic frameworks (MOFs), covalent organic frameworks (COFs) [47–49], and semiconductor systems such as silver vanadate [1, 2, 50], as well as through the development of hybrid NPs (Se-AgNPs) [4–13]. These hybrid nanostructures exhibit high physicochemical stability and enable sustained ion release, which synergistically contributes to the elimination of planktonic microorganisms and biofilms [1, 4, 47].

While previous reviews have addressed AgNPs or SeNPs individually, and some have covered hybrid NP broadly (including synthesis methods and general applications), no systematic review has specifically focused on Se-AgNPs. Given the promising evidence that combining AgNPs with SeNPs can enhance antimicrobial efficacy while reducing cytotoxicity, this systematic review aims to address the following research question: “Can the addition of SeNPs to AgNPs enhance their antimicrobial and biological properties?”. This review compiles evidence on Se-AgNPs, highlighting their synergistic antimicrobial activity and the cytoprotective role of SeNPs in mitigating cytotoxicity. By providing a focused, evidence-based synthesis, this review fills a clear gap in the literature and offers a novel perspective on the biomedical applications of Se-AgNPs, including the potential development of new products and infection-control strategies.

## Materials and Methods

### Registration, Protocol, and Formulating the Review Question

This review was registered in the Open Science Framework (OSF, osf.io/2h95e) and structured according to the Preferred Reporting Items for Systematic Reviews and Meta-Analyses (PRISMA) 2020 checklist [51].

The review question “Can the addition of SeNPs to AgNPs enhance their antimicrobial and biological properties?” was formulated based on the PICOS strategy, which included: population (microbial strains, biofilm models, or mammalian cell lines), intervention (exposure to Se-AgNPs), comparison (pure SeNPs and AgNPs), outcomes (primary: antimicrobial activity against planktonic microorganisms and biofilms; secondary: biological activity including cytotoxicity, genotoxicity, and mutagenicity), and study design (in vitro).

### Databases and Search Strategy

Keywords were selected using Medical Subject Headings (MeSH) terms and combined with Boolean operators (AND, OR). Searches were conducted in the Scopus, PubMed, Embase, LILACS, ScienceDirect, Google Scholar, and ProQuest databases using the following strategy: ((“Selenium” OR “SeNPs”) AND (“Silver” OR “Silver nanoparticles” OR “Ag” OR “AgNPs”) AND (“Antimicrobial” OR “Microbial” OR “Antibiofilm” OR “Antibacterial” OR “Antifungal” OR “Biological” OR “Biocompatibility” OR “Cytotoxicity” OR “Genotoxicity” OR “Mutagenicity” OR “Cell **t**oxicity”)). All searches were conducted on a single date, November 15, 2024. For Google Scholar and ProQuest, the first 150 results were screened. Database-specific search adaptations are detailed in Supplementary Table 1. No language or publication date restrictions were applied.

Study selection was conducted in two stages, both independently performed by two reviewers (J.M.C.S and A.C.R). Disagreements were resolved by consensus between the reviewers. In the first phase, duplicate articles were removed using EndNote (Clarivate Analytics, UK), followed by title and abstract screening in Rayyan (Qatar Computing Research Institute, Qatar) [52]. Studies that did not meet the predefined inclusion and exclusion criteria were excluded. In Phase two, the full texts of the remaining articles were assessed for eligibility.

### Inclusion and Exclusion Criteria

The inclusion criteria focused on in vitro experimental studies assessing the antimicrobial and biological activities of Se-AgNPs. For antimicrobial activity, studies evaluating effects against bacteria and fungi in both planktonic and biofilm forms were included. The methods used were minimum inhibitory concentration (MIC), agar diffusion assay, colony-forming units (CFU) counts, and microbial viability analysis via spectrophotometry. For biological activity, studies assessing cytotoxicity based on cell viability measured by spectrophotometry were included. No studies were identified that evaluated the genotoxicity or mutagenicity of Se-AgNPs.

Exclusion criteria included book chapters, systematic reviews, clinical trials, letters to the editor, conference abstracts, and short communications. Studies that did not evaluate the effect of SeNPs incorporation into AgNPs on antimicrobial and biological activities were excluded, as well as those combining Se-AgNPs with other compounds or using AgNP-based materials at the micrometric scale. (Supplementary Table 2).

### Data Extraction and Synthesis Strategy

Qualitative data on in vitro antimicrobial and biological activity of Se-AgNPs were independently extracted by 2 reviewers (J.M.C.S and A.C.R) using a standardized table in an online processing program (Google Docs, Google) (Tables 1 and 2). The extracted variables included author and year, AgNPs, SeNPs, control groups, microorganisms, cell types, methods, results, and conclusions.

**Table 1:** Antimicrobial activity of the combination of selenium and silver nanoparticles (Se-AgNPs)

Author and year	AgNPs	SeNPs	Comparison	Microorganisms	Methods	Results	Conclusion
Ahmad et al. [4]	1. AgNPs. **Shape**: Spherical. **Size**: 50–100 nm; **Concentrations**: 250, 500, 1000, and 2000 µg/mL.	2. SeNRs. **Size**: 300–400 nm; **Concentrations**: 250, 500, 1000, and 2000 µg/mL.	3. Ag-SeNRs; **Size**: combination of the above; **Concentrations**: 250, 500, 1000, and 2000 µg/mL. 4. Ampicillin (positive control).	*Escherichia* *coli* and *Bacillus subtilis*.	1. MIC assay (0.5 McFarland turbidity); 2. Agar diffusion assay.	1. The MIC for both microorganisms was similar for AgNPs and SeNRs, ranging from 30 to 45 µg/mL. A lower MIC (15 µg/mL), comparable to the positive control, was observed for the combined Ag-SeNRs. 2. Ampicillin showed an inhibition zone of 23 mm for both microorganisms. Against *E. coli* and *B. subtilis*, respectively, SeNRs exhibited inhibition zones ranging from 3–8 mm and 6–14 mm; AgNPs showed 8–15 mm and 9–17 mm; and Ag–SeNRs demonstrated 3–30 mm and 13–20 mm.	The synergistic combination of Ag and Se in the Ag-SeNRs composite resulted in superior antimicrobial activity, outperforming ampicillin, AgNPs, and SeNRs. All nanomaterials demonstrated concentration-dependent activity.
Attia et al. [5]	1. AgNPs. **Size**: N/I. **Concentration**: 100 µg/mL.	2. SeNPs. **Size**: N/I. **Concentration**: 100 µg/mL.	3. Se-AgNPs. **Size**: N/I. **Concentration**: 100 µg/mL.	*Ralstonia solanacearum.*	Agar diffusion assay.	Se-AgNPs exhibited the largest inhibition halo (27.5 ± 1.8 mm), followed by AgNPs (17.9 ± 0.96 mm) and SeNPs (14.5 ± 1.32 mm).	The synergistic activity of Se-AgNPs resulted in a larger inhibition halo compared to the individual nanomaterials. AgNPs presented higher efficacy than SeNPs.
Delgado-Beleño et al. [6]	1. AgNPs. **Size**: 4.2 nm; **Concentration**: 20.000 µg/mL.	2. Ag_2_Se. **Size**: 9 nm; **Concentration**: 20.000 µg/mL	3. Gentamicin (positive control).	*Staphylococcus aureus*,* Streptococcus agalactiae*,* E. coli*, and *Klebsiella pneumoniae.*	Agar diffusion assay.	The following inhibition zone diameters were observed for AgNPs, Ag_2_SeNPs, and gentamicin, respectively: *S. aureus* (21.5, 17, and 19 mm), *S. agalactiae (*14, 12, and 0 mm), *E. coli* (10.5, 10.5, and 9 mm), and *K. pneumoniae (*12.5, 11, and 10 mm).	AgNPs demonstrated higher antimicrobial activity compared to the Ag-Se-based compound, which in turn exhibited superior activity relative to the positive control.
Elakraa et al. [7]	1. AgNPs. **Size**: 10.95 nm; **Concentration**: 20.000 µg/mL.	2. SeNPs. **Size**: 20.54 nm; **Concentration**: 20.000 µg/mL.	3. Se-AgNPs. **Size**: 12.69 nm; **Concentration**: 20.000 µg/mL.	*E. coli*,* Pseudomonas aeruginosa*,* K. pneumoniae* (Gram-negative), *S. aureus*,* Enterococcus* spp. (Gram-positive), and *Candida albicans* (fungal strain).	1. MIC assay; 2. Agar diffusion assay; 3. Antibiofilm evaluation (0.5 McFarland turbidity) by spectrophotometry.	1. All nanomaterials exhibited similar MIC = 2.5 µg/mL; 2. The observed diameters for AgNPs, SeNPs, and Se-AgNPs were, respectively: *S. aureus* (12, 19, and 17 mm), *E. coli* (21, 22, and 20 mm), *P. aeruginosa* (18, 9, and 20 mm), *Enterococcus* spp. (13, 11, and 16 mm), *K. pneumoniae* (18, 16, and 17 mm), and *C. albicans* (17, 26, and 15 mm); 3. Se-AgNPs showed a reduction ranging from 74.93% to 91.88% across all microbial biofilms tested.	All NPs demonstrated high microbial sensitivity. Se-AgNPs exhibited strong antibiofilm activity. AgNPs were more effective against Gram-negative bacteria, while SeNPs showed superior activity against fungal strains and certain Gram-positive bacteria. Se-AgNPs, despite their synergistic action, presented variable and intermediate antimicrobial effects depending on the specific pathogen targeted.
El-Behery et al. [8]	1. AgNPs. **Size**: 34.3 nm; **Concentrations**: (62.5, 125, 250, 500, and 100 µg/mL).	2. SeNPs. **Size**: 40.7 nm; **Concentrations**: (62.5, 125, 250, 500, and 100 µg/mL).	3. Se-AgNPs. **Size**: 46.7 nm; **Concentrations**: (62.5, 125, 250, 500, and 100 µg/mL). 4. Nystatin (fungal strains); 5. Amoxicillin + clavulonic acid (bacterial strains).	*E. coli*, *K. pneumoniae*, *P. aeruginosa* (Gram-negative), *Bacillus cereus*,* Bacillus subtilis* (Gram-positive), *Aspergillus brasiliensis*,* C. albicans*,* Alternaria alternata*, and *Fusarium oxysporum* (fungal strains).	1. Agar diffusion assay; 2. Antibiofilm evaluation (0.5 McFarland turbidity) by spectrophotometry.	1. All tested microorganisms showed dose-dependent inhibition. For fungal strains, AgNPs and SeNPs exhibited inhibition zones ranging from 14 to 32.7 mm and 14 to 35 mm, respectively, whereas Se-AgNPs demonstrated higher activity, ranging from 11.3 to 42.7 mm. For bacterial strains, AgNPs, SeNPs, and Se-AgNPs showed inhibition zones of 12.7–34.3 mm, 8.7–38.7 mm, and 7.3–34.7 mm, respectively; 2. AgNPs inhibited biofilm formation by 56–87.6%, SeNPs by 50.7–86.4%, and Ag–Se NPs achieved the highest inhibition, ranging from 77.8–90.9%, across *E. coli*,* S. aureus*,* P. aeruginosa*,* K. pneumoniae*, and *C. albicans.*	The synergy between Ag and Se in Se-AgNPs resulted in enhanced microbial sensitivity and antibiofilm activity. SeNPs exhibited a larger inhibition halo, while AgNPs demonstrated stronger antibiofilm efficacy.
Hashem et al. [9]	1. AgNO_3_. **Size**: N/I; **Concentration**: 100 µg/mL.	2. Na_2_SeO_3_. **Size**: N/I; **Concentration**: 100 µg/mL.	3. Se-AgNPs. **Size**: 24.5 nm; **Concentration**: 100 µg/mL. 4. Ampicillin-Sulbactam.	*C. albicans* (fungal strain), *E. coli*,* P. aeruginosa*,* Klebsiella oxytoca* (Gram-negative), *B. subtilis*, and *S. aureus* (Gram-positive).	1. MIC assay; 2. Agar diffusion assay.	1. A lower MIC (12.5–50 µg/mL) was observed for Se-AgNPs compared to ampicillin-sulbactam for all tested microorganisms, whereas AgNO_3_ and Na_2_SeO_3_ showed no activity; 2. No inhibition zones were observed for AgNO_3_ and Na_2_SeO_3_, whereas Se-AgNPs exhibited antibacterial activity against *E. coli*,* P. aeruginosa*,* B. subtilis*, and *S. aureus*, with inhibition zones of 14.33 ± 0.58, 18.87 ± 0.76, 16.17 ± 0.76, and 17.17 ± 1.26 mm, respectively. Se-AgNPs also showed weak antifungal activity against *C. albicans*, with an inhibition zone of 12.27 ± 1.42 mm.	The synthesized precursors of Se-AgNPs did not show antimicrobial activity; however, Se-AgNPs were more effective than ampicillin-Sulbactam.
Li et al. [10]	-	1. SeNPs. **Size**: N/I; **Concentrations**: 0, 6, 12, 24, 36, and 48 µg/mL.	2. Se-AgNPs (Ag encapsulated by Se). **Size**: 85 nm; **Concentrations**: 0, 6, 12, 24, 36, and 48 µg/mL.	*S. aureus* (Gram-positive) and *E. coli* (Gram-negative).	1. MIC assay; 2. Antibiofilm activity (CFU count); 3. Antibiofilm activity (violet crystal and ROS assays) with an inoculum of 1 × 10^5^ CFU.	1. A lower MIC (50 µg/mL) was observed for Se-AgNPs compared to SeNPs (800 µg/mL). 2. The following CFU counts were observed for *S. aureus* and *E. coli*, respectively: control (1.3 × 10^6^ and 1.6 × 10^6^), SeNPs (2 × 10^5^ and 9 × 10^5^), and Se–AgNPs (1 × 10^5^ and 4 × 10^5^). 3. A reduction in biofilm formation was observed for *S. aureus* and *E. coli*, respectively: Se-AgNPs (80% and 40%) and SeNPs (65% and 15%), proportional to the increase in ROS production.	The synthesis of Se-AgNPs enhanced the antibiofilm efficacy of SeNPs. The effectiveness of the NPs was concentration-dependent.
Liang et al. [11]	1. Surface coated with AgNPs. **Size**: 10 nm.	2. SeNPs. **Size**: 40–70 nm.	2. Surface coated with Se-AgNPs **Size**: 600–1500 nm.	*S. aureus* (Gram-positive) and *E. coli* (Gram-negative).	1. Antibacterial activity against planktonic strains; 2. Anti-adhesion activity (crystal violet) with 2 × 10^6^ CFU inoculum.	1. Microbial reduction was observed at 24 h and 72 h, respectively, for AgNPs (OD = 0.7 and 0.8) and Se-AgNPs (OD = 0.8 and 1.1). 2. Surfaces coated with Se-AgNPs showed reduced microbial adhesion and biofilm formation, with biofilm reductions of 64.7% and 30.3% at 24 h, and 81.2% and 59.7% at 72 h for *S. aureus* and *E. coli*, respectively, compared to AgNP-coated surfaces.	Surfaces coated with AgNPs and Se-AgNPs were effective against planktonic bacteria for up to 72 h, with AgNPs showing better results. However, Se-AgNPs were more effective at inhibiting microbial adhesion and biofilm formation compared to AgNPs.
Ozdal, [12]	1. AgNPs. **Size**: 10–15 nm; **Concentrations**: 50, 100, and 200 µg/mL.	2. SeNPs. **Size**: 20–30 nm; **Concentrations**: 50, 100, and 200 µg/mL.	3. Ag_2_SeNPs. **Size**: 30–40 nm; **Concentrations**: 50, 100, and 200 µg/mL.	*S. aureus* (Gram-positive) and *E. coli* (Gram-negative).	1. MIC assay; 2. Antibiofilm evaluation (0.5 McFarland turbidity) by spectrophotometry.	1. For *E. coli*, the lowest MIC was observed for AgNPs, Ag_2_SeNPs, and SeNPs. For *S. aureus*, the lowest MIC was observed for Ag_2_SeNPs, followed by AgNPs and SeNPs, with all MIC values ranging between 150 and 250 µg/mL. 2. Biofilm inhibition was observed for *E. coli* and *S. aureus*, respectively: AgNPs (97% and 94%), SeNPs (90% and 84%), and Ag_2_SeNPs (97% and 99%).	The combination of Se and Ag in Ag_2_SeNPs resulted in enhanced antimicrobial activity, with AgNPs contributing more strongly than SeNPs.
Yang et al. [13]	1. AgNPs. **Size**: 18 ± 2.6 nm; **Concentrations**: 0, 10, 20, 30, 40, and 50 µg/mL.	2. SeNPs (nanowires). **Size**: 122 ± 8.5; **Concentrations**: 0, 10, 20, 30, 40, and 50 µg/mL.	3. Se-AgNPs (Se nanowires with AgNPs). **Size**: 148 ± 11.2 nm; **Concentrations**: 0, 10, 20, 30, 40, and 50 µg/mL. 4. Ampicillin (positive group).	*S. aureus* (Gram-positive) and *E. coli* (Gram-negative).	1. MIC assay; 2. Antibiofilm activity (CFU count); 3. Live/Dead assay; 4. Evaluation of the mechanism of action (ROS and bacterial integrity).	1. The lowest MICs for *S. aureus* and *E. coli* were observed for Se-AgNPs (28.7 and 36.8 µg/mL), followed by AgNPs (42.1 and 52.4 µg/mL) and SeNPs (65.5 and 82 µg/mL). Ampicillin showed the highest MIC values (> 128 µg/mL); 2, 3, and 4. For both microorganisms, Se-AgNPs showed the highest efficacy (98% reduction), followed by AgNPs (85% reduction) and SeNPs (20%). Efficacy was dependent on time and concentration, with concentrations of 40 and 50 µg/mL showing similar results.	The SeNPs nanowires showed lower antimicrobial efficacy. However, when AgNPs were added to their surface, the Se-AgNPs composite exhibited high antimicrobial and antibiofilm activity. The mechanisms of action were related to the formation of ROS and the destruction of the integrity of microorganisms.

*MIC* Minimum inhibitory concentration, *Ag* Silver, *Se* Selenium, *AgNPs* Silver nanoparticles, *SeNPs* Selenium nanoparticles, *SeNRs* Selenium nanorods, *N/I* Not informed, *Ag*_*2*_*Se* silver selenide, *Na*_*2*_*SeO*_*3*_ sodium selenite, *AgNO*_*3*_ silver nitrate, *CFU* Colony Forming Units, *ROS* Reactive Oxygen Species

**Table 2:** In vitro biological activity of the combination of Selenium and Silver nanoparticles (Se-AgNPs)

Author and year	AgNPs	SeNPs	Comparison	Cells	Methods	Results	Conclusion
Hashem et al. [9]	N/I.	N/I.	3. Se-AgNPs. **Size**: 24.5 nm; **Concentration**: 7.81, 15.62, 31.25, 62.5, 125, 250, 500, and 1000 µg/mL.	Normal human fibroblast cell line (Wi38) and mama cancerous cell line (MCF7).	Cytotoxicity by MTT assay.	A concentration of up to 168.42 µg/mL showed no cytotoxicity against Wi38 cells. However, concentrations ranging from 250 to 1000 µg/mL resulted in a 90% inhibition of cell viability. For MCF7 (breast cancer) cells, the safe concentration of 125 µg/mL was effective in inhibiting 90.3% of the cancer cells.	Se-AgNPs showed low toxicity when used up to 168.42 µg/mL, and they exhibited high anticancer activity up to the same concentration.
Liang et al. [11]	1. Surface coated with AgNPs. **Size**: 10 nm.	2. SeNPs **Size**: 40–70 nm.	2. Surface coated with Se-AgNPs. **Size**: 600–1500 nm.	Human fetal osteoblasts.	1. Cytotoxicity (cell viability by violet cristal); 2. Ion release (ICP).	1. The surface coated with AgNPs showed 51.7% inhibition of cell proliferation, while the surface containing Se-AgNPs showed 85.9% proliferation; 2. Higher control of ion release was observed for Se-AgNPs compared to AgNPs.	The addition of SeNPs to the AgNPs coating optimized the surface, allowing for higher cell viability and proliferation, likely due to the controlled release of ions.

*Ag* Silver, *Se* Selenium, *AgNPs* Silver nanoparticles, *SeNPs* Selenium nanoparticles, *N/I* Not informed, *Ag*_*2*_*Se* silver selenide, *Na*_*2*_*SeO*_*3*_ sodium selenite, *AgNO*_*3*_ silver nitrate

Due to substantial methodological heterogeneity across the available evidence, including differences in NP properties, experimental models, exposure conditions, outcome measures, and lack of comparable control groups, a meta-analysis was not feasible. The limited number of studies also precluded subgroup and sensitivity analyses, and prevented the definition of a common effect measure for quantitative synthesis. Therefore, data were synthesized narratively, focusing on consistent trends across studies.

### Risk of Bias

Risk of bias was assessed using the Joanna Briggs Institute (JBI) Critical Appraisal Checklist adapted for in vitro studies [53] and analyzed using RevMan Web (Cochrane). Two independent reviewers (J.M.C.S. and A.C.R.) evaluated all included studies across six methodological domains: clarity of cause and effect (D1), similarity of comparison groups (D2), similarity of treatment conditions apart from the intervention of interest (D3), presence of an appropriate control group (D4), consistency and reliability of outcome measurement (D5), and appropriateness of statistical analysis (D6).

The JBI tool does not generate an overall risk-of-bias score; instead, it assesses methodological quality at the level of individual domains, with each domain evaluated independently. Each domain was classified as “low risk”, “high risk”, or “unclear risk” of bias. Low risk was assigned when sufficient methodological information indicated adequate experimental design and reporting. High risk was assigned when methodological limitations potentially affecting validity or reproducibility were identified. Unclear risk was assigned when insufficient or ambiguous methodological information prevented a reliable judgment. Disagreements between the two reviewers were resolved through discussion and consensus.

## Results

### Search Results and Studies Included

**Fig. 1:**
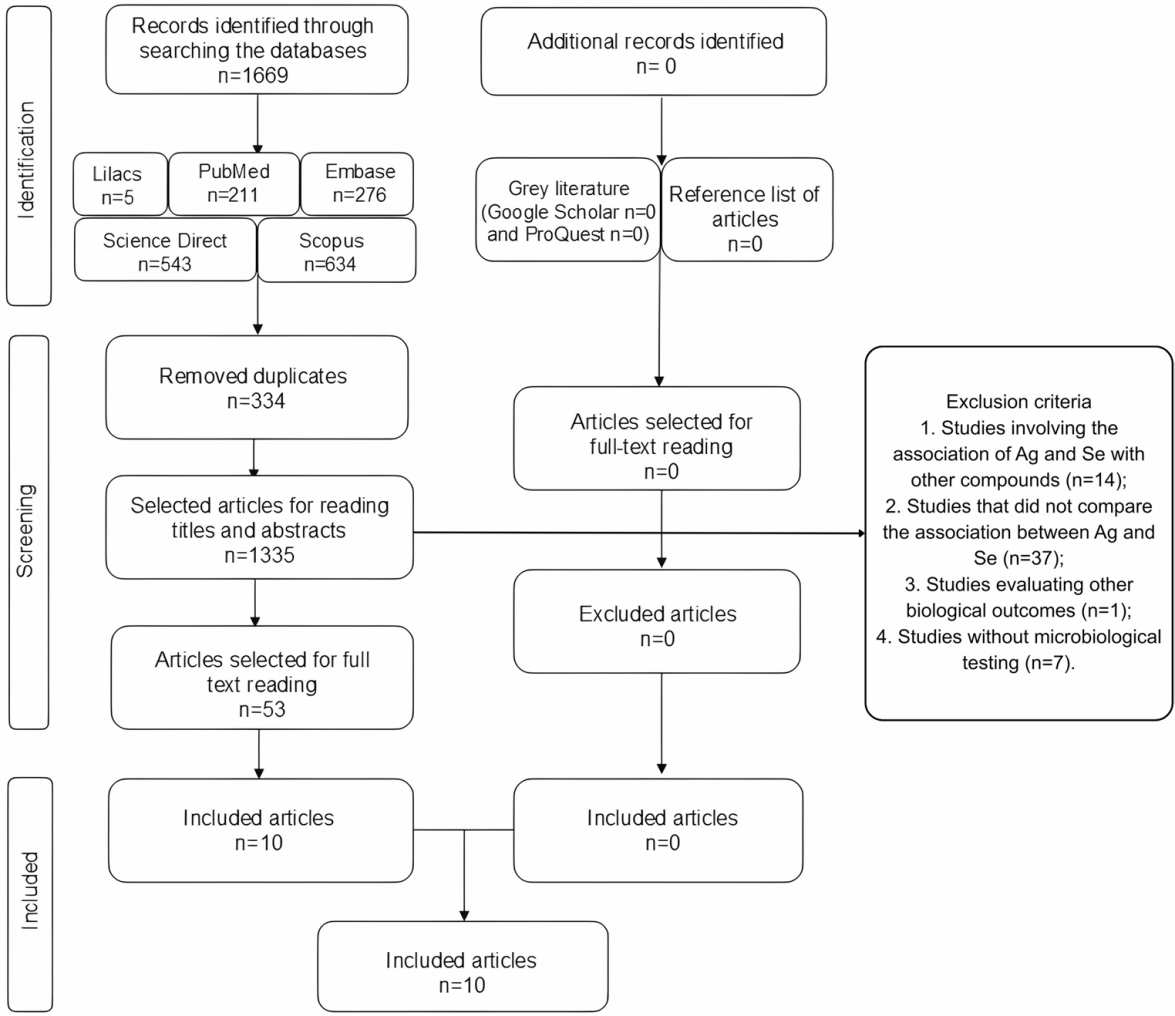
Flowchart of the process of including studies in the systematic review

A total of 211 articles were retrieved from PubMed, 543 from ScienceDirect, 276 from Embase, 634 from Scopus, 5 from LILACS, with no records identified in Google Scholar or ProQuest during the November-December 2024 search period. Of the 1,669 articles initially identified, 334 duplicates were removed using EndNote and Rayyan. The remaining 1,335 studies were screened by title and abstract, of which 53 articles were selected for full-text assessment. Ultimately, 10 studies met the eligibility criteria and were included in this systematic review [4–13] (Fig. 1).

### Risk of Bias

The independent assessment of risk of bias between reviewers demonstrated strong inter-rater agreement (Kappa = 0.87). Among the 10 included studies, domain D4 (presence of control groups) showed a high risk of bias in 30% of the studies due to the absence of positive or negative control groups for comparative analyses [5, 9, 12]. Regarding domain D6 (appropriateness of statistical analysis), 60% of the studies were classified as high risk due to the absence of statistical analyses [4–7, 9, 12], and 20% were classified as unclear risk due to insufficient transparency regarding the methods employed [10, 13]. All other domains were assessed as having a low risk of bias (Table 3).

**Table 3:** Risk of bias assessment of included studies according to the JBI tool

Author and year	D1	D2	D3	D4	D5	D6
Ahmad et al. [4]	LOW	LOW	LOW	LOW	LOW	HIGH
Attia et al. [5]	LOW	LOW	LOW	HIGH	LOW	HIGH
Delgado-Beleño et al. [6]	LOW	LOW	LOW	LOW	LOW	HIGH
Elakraa et al. [7]	LOW	LOW	LOW	LOW	LOW	HIGH
El-Behery et al. [8]	LOW	LOW	LOW	LOW	LOW	LOW
Hashem et al. [9]	LOW	LOW	LOW	HIGH	LOW	HIGH
Li et al. [10]	LOW	LOW	LOW	LOW	LOW	UNCERTAIN
Liang et al. [11]	LOW	LOW	LOW	LOW	LOW	LOW
Ozdal, [12]	LOW	LOW	LOW	HIGH	LOW	HIGH
Yang et al. [13]	LOW	LOW	LOW	LOW	LOW	UNCERTAIN

D means domain. D1: is it clear in the study what the cause is and what the effect is? D2: were the participants included in any comparison similar? D3: were the participants included in any comparison receiving similar treatment/care other than the exposure or intervention of interest? D4: was there a control group? D5: were the outcomes of participants included in any comparison measured in the same way? D6: was appropriate statistical analysis used?

### Characteristics of NPs

The studies investigated AgNPs ranging in size from 3.7 to 100 nm, SeNPs from 9 to 400 nm, and Se-AgNPs from 12.59 to 400 nm, with concentrations ranging from 6 to 20,000 µg/mL [5–13]. The NPs exhibited spherical [5–12], nanobasket [4], and nanowire morphology types [13]. Additional Se-AgNPs [6, 12] and SeNP-based compounds [9], specifically silver selenide (Ag_2_Se) and sodium selenite (Na_2_SeO_3_), respectively, were also used.

### Antimicrobial Effect of SeNPs and AgNPs

Antimicrobial activity against planktonic strains and their sensitivity to NPs was assessed using MIC [4, 7, 9, 10, 12, 13], agar diffusion [4–9], and cell viability in suspension assays [11]. The Gram-positive bacteria investigated included *Bacillus subtilis* [4, 8, 9], *Bacillus cereus* [8], *S. aureus* [6–13], *Streptococcus agalactiae* [6], and *Enterococcus* spp [7]. Gram-negative strains included *Escherichia coli* [4, 6–13], *Ralstonia solanacearum* [5], *Klebsiella pneumoniae* [6–8], *P. aeruginosa* [7–9], and *Klebsiella oxytoca* [9]. Fungal strains such as *C. albicans* [7–9], *Aspergillus brasiliensis* [8], *Alternaria alternata* [8], and *Fusarium oxysporum* [8] were also evaluated.

Se-AgNPs exhibited lower MIC [4, 9, 10, 13] and larger zones of inhibition [4, 5, 8] compared to AgNPs and SeNPs. However, Elakraa et al. [7] reported similar MIC values and zones of inhibition among the **NPs** tested. In contrast, Ozdal et al. [12] found that AgNPs had a lower MIC than the Ag_2_Se compound, while SeNPs demonstrated higher MIC overall. Additionally, compounds such as Ag_2_Se [6], AgNO_3_, and Na_2_SeO_3_ [9] exhibited minimal or no formation of zones of inhibition. In suspension assays with planktonic bacteria, Teflon coated with AgNPs was more effective at reducing microbial viability than coatings with Se-AgNPs [11].

For all NPs, antimicrobial efficacy was found to be concentration-dependent [4]. Ampicillin demonstrated higher efficacy than both AgNPs and SeNPs, whereas Se-AgNPs outperformed them [4, 9, 13]. The Ag_2_Se compound was reported to be more effective than gentamicin [6]. In contrast, nystatin and amoxicillin were less effective than all NP formulations tested [8].

### Antibiofilm Effect of SeNPs and AgNPs

The antibiofilm activity of NPs was evaluated using monospecies biofilm models of *E. coli* [7, 8, 10–13], *P. aeruginosa* [7, 8], *K. pneumoniae* [7, 8], *S. aureus* [7, 8, 10–13], *Enterococcus* spp [7]., and *C. albicans* [7, 8]. Microorganisms were standardized using 0.5 McFarland turbidity [7, 8, 12, 13], 1 × 10^6^ CFU/mL [10], and 2 × 10^6^ CFU/mL [11]. Biofilm incubation periods varied, including overnight [7, 8], 24 h [11–13], 48 h [10], and 72 h [11].

Variations in concentrations of NPs were reported across studies, ranging from 5.0 µg/mL [8], 10 µg/mL [7], and 24 µg/mL [10] to higher concentrations between 50 and 200 µg/mL [12] and 512 µg/mL [13]. Different methods were employed to assess antibiofilm activity, including crystal violet staining followed by spectrophotometric quantification [7, 8, 10–12], CFU counting [10, 13], and fluorescence microscopy using Live/Dead staining [13].

Se-AgNPs exhibited superior antibiofilm activity compared to AgNPs and SeNPs [7, 8, 10, 13], with effectiveness shown to be concentration-dependent [12]. The Ag_2_Se also demonstrated high antibiofilm efficacy when compared to pure AgNPs and SeNPs [12]. Teflon surfaces coated with Se-AgNPs showed reduced microbial adhesion and biofilm formation at 24 h; however, biofilm formation increased after 72 h [11]. Furthermore, two studies attributed the enhanced antibiofilm activity to increased ROS generation [10, 13], and 1 study reported damage to microbial membranes as a potential mechanism [13].

### Biological Activity of SeNPs and AgNPs

No studies were found that specifically evaluated the genotoxic or mutagenic effects of the association between AgNPs and SeNPs. Only two studies investigated the cytotoxicity of Se-AgNPs, 1 study using pure NPs [9] and the other evaluating Se-AgNPs coated onto a Teflon (PTFE) surface [11]. The NPs ranged in size from 24.5 to 1500 nm [9, 11], and the concentrations tested varied from 7.81 to 1000 µg/mL [9].

Methodological differences were noted between the studies. Hashem et al. [9] used human fibroblasts (Wi38) and assessed cell viability using the MTT assay, whereas Liang et al. [11] employed human osteoblasts and evaluated viability using crystal violet staining. Despite the methodological variations, the Se-AgNPs exhibited low cytotoxicity [9]. In addition, they promoted high levels of cell adhesion, viability, and proliferation [11].

## Discussion

**Fig. 2:**
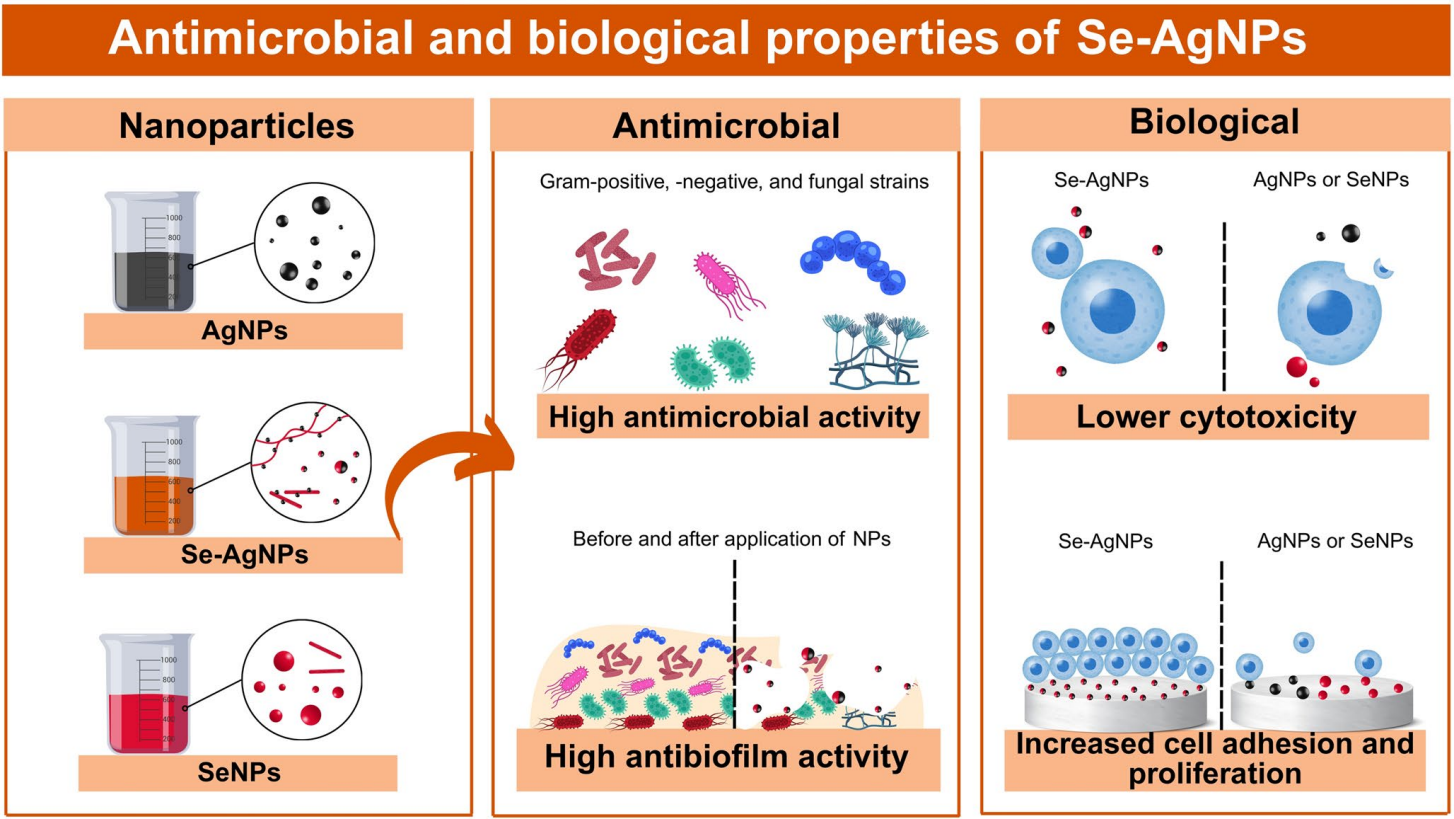
Graphical abstract. Antimicrobial and biological properties of Se-AgNPs

The studies included in this systematic review suggest that Se-AgNPs exhibit enhanced antimicrobial activity and reduced cytotoxicity (Fig. 2) [4–13]. However, these findings should be interpreted cautiously due to the limited number of studies and substantial methodological heterogeneity, including variations in NPs properties, experimental design, control groups, and evaluation protocols, which limit interstudy comparability. Similar limitations have been reported in previous systematic reviews of AgNPs and SeNPs, identifying poor methodological standardization and limited experimental reproducibility as major barriers to robust evidence synthesis and clinical translation [3, 14, 15, 19, 38]. Consequently, clinically relevant safety and efficacy thresholds for NP-based antimicrobial systems remain poorly defined.

 Robust evidence synthesis and future meta-analytical validation will require standardized methodological frameworks across studies. Harmonization of characterization of NPs, microbial and biofilm models, control conditions, exposure protocols, and validated toxicological endpoints is essential to improve reproducibility and enable meaningful interstudy comparison. Such standardization would also strengthen regulatory evaluation and facilitate the clinical translation of Se-AgNP-based systems for infection control. Despite these limitations, the available evidence consistently supports a synergistic improvement in antimicrobial efficacy and a reduction in reduced cytotoxicity of Se-AgNPs [4–13].

**Fig. 3:**
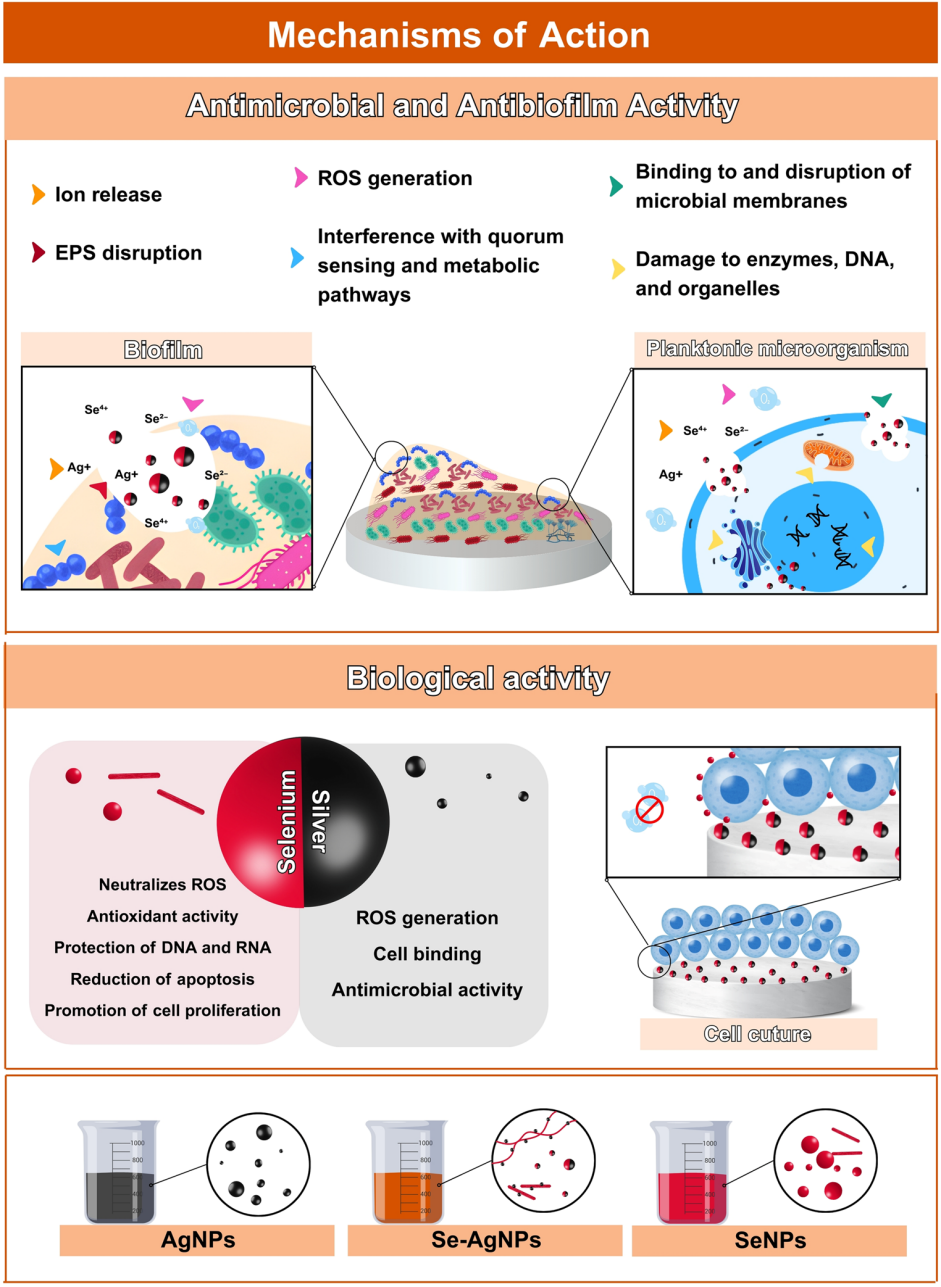
Antimicrobial and biological mechanism of action of Se-AgNPs

 Se-AgNPs demonstrate broad-spectrum antimicrobial activity, likely driven by synergistic interactions between AgNPs and SeNPs components (Fig. 3) [4–13]. This synergism enhances antimicrobial activity while reducing cytotoxic effects compared with isolated NPs, suggesting a more favorable therapeutic window [9, 13]. The antimicrobial activity of AgNPs is attributed to the release of Ag^+^, which binds electrostatically to cell membranes, disrupts glycoproteins and lipids, generates ROS, and causes damage to cellular enzymes and DNA [1–5, 14, 15]. SeNPs enhance this mechanism through their selenite (Se^2−^) and selenate (Se^4+^) ions, which interfere with sulfur-containing biomolecules, such as sulfhydryl groups in membranes, enzymes, and microbial DNA [7–9, 41, 54–56]. These combined mechanisms likely explain the enhanced antimicrobial performance and metabolic disruption observed with Se-AgNPs [7–9, 41, 54–56] (Fig. 2).

 Beyond planktonic activity, AgNPs and SeNPs also target biofilm-associated phenotypes by impairing microbial adhesion and compromising EPS integrity [7, 8, 10–13]. This effect is driven by oxidative stress and ion-mediated interactions (Ag^+^, Se^2−^, and Se^4+^), which induce structural and functional damage to the EPS matrix [7, 8, 10–13]. These ions further interfere with key metabolic pathways and quorum sensing signaling, disrupting biofilm coordination [1–3, 7, 8, 10–13]. Collectively, these mechanisms weaken biofilm resilience, enhance nanoparticle susceptibility, and may facilitate host immune clearance.

 AgNPs exhibit stronger antibacterial activity against Gram-positive and Gram-negative bacteria, whereas SeNPs show relatively higher antifungal activity [4–13]. These differences reflect the distinct structural features of microbial cell walls and their varying susceptibility to NPs [15]. AgNPs and released Ag^+^ act directly on the thick peptidoglycan-rich cell walls of Gram-positive bacteria and the thinner, lipopolysaccharide-rich (LPS) outer membranes of Gram-negative bacteria [15]. Both bacterial types are highly susceptible to Ag^+^ ion penetration, membrane disruption, and ROS generation, which leads to microbial death [4–13].

In contrast, the reduced antibacterial activity of SeNPs is attributed to the slow release of Se^2−^ ions, which limits their interaction with sulfur-containing proteins in microbial membranes [4, 9, 13]. This, in turn, reduces their ability to disrupt microbial metabolism and induce microbial death [4, 9, 13]. However, fungal strains such as *C. albicans*, characterized by larger and more rigid cell walls composed of chitin, glucans, and ergosterol (structures that confer resistance to AgNPs) exhibit higher susceptibility to SeNPs [54]. This susceptibility is associated with the ability of SeNPs to inhibit fungal dimorphism, reduce virulence, and exert fungicidal effects [54].

 NPs have been reported to exhibit enhanced antimicrobial performance compared with conventional agents, largely due to their broad-spectrum and multitarget mechanisms, which simultaneously affect microbial glycoproteins, lipids, and nucleic acids [1–3, 7, 8, 10–13]. In contrast, conventional antibiotics typically act through highly specific targets, such as inhibition of bacterial cell wall synthesis by β-lactams (e.g., ampicillin and amoxicillin), disruption of protein synthesis via the 30 S ribosomal subunit by aminoglycosides (e.g., gentamicin), or ergosterol binding in fungal membranes by polyenes (e.g., nystatin), leading to membrane permeabilization and cell death [57–60]. From a translational perspective, this mechanistic contrast highlights the broader antimicrobial scope of NPs, which may contribute to reduced susceptibility to conventional resistance pathways and support their potential role in addressing antimicrobial resistance.

 Se-AgNPs may improve cell viability and reduce cytotoxicity primarily through the antioxidant activity of Se ^2−^ and Se^4+^ ions, which counterbalance ROS generation induced by AgNPs [42–45]. Selenium is a key component of antioxidant defense systems, including enzymes such as GSH-Px and TrxR, thereby mitigating oxidative damage to cellular components [42–45]. This redox modulation contributes to improved cellular survival and reduced apoptosis in exposed cells [42–45]. In addition, SeNPs has been associated with the regulation of signaling pathways involved in tissue repair, including VEGF and TGF-β, suggesting a potential role in supporting regenerative processes [11, 35, 36, 61, 62].

 Modulation of ROS levels may contribute to limiting oxidative damage to the extracellular matrix (ECM) in infected tissues [9, 11]. SeNPs may interact with extracellular matrix components, such as collagen and fibronectin, potentially influencing cell-matrix interactions [9, 11]. SeNPs also stimulate the expression of integrins and transmembrane proteins, which activate intracellular signaling pathways, including PI3K/Akt/mTOR, involved in cell cycle regulation and tissue regeneration [11, 35, 36, 61, 62]. Additionally, SeNPs has been suggested to influence the biotransformation of AgNPs through selenoprotein-related pathways, potentially reducing their bioavailability [11, 35, 36]. These mechanisms may contribute to reduced AgNPs bioavailability and potentially mitigate cytotoxic effects [11, 35, 36].

 Ag _2_ Se exhibits lower antimicrobial activity compared to AgNPs, likely due to its higher chemical stability, which limits Ag ^+^ and Se^2−^ ions release and associated ROS generation [12]. This may result in reduced interaction with microbial structures and attenuated effects on biofilm and metabolic activity [12]. In contrast, AgNPs show higher reactivity to environmental conditions such as oxygen and light, which may enhance Ag^+^ release and antimicrobial activity [15]. However, increased ion release may also be associated with higher cytotoxicity, potentially limiting the safe application of AgNPs in human tissues [15].

Se-AgNPs exhibit promising antimicrobial activity with low cytotoxicity; however, their genotoxic and mutagenic potential requires further investigation. No included studies evaluated DNA damage, chromosomal alterations, or mutagenicity using standardized assays such as comet or micronucleus tests. This is particularly relevant given that NPs may interact with genetic material through oxidative stress, ion release, and intracellular accumulation. SeNPs may exert protective effects and mitigate AgNP-induced cytotoxicity; however, its long-term genomic safety remains unverified. In the absence of direct genotoxic assessments, the overall genomic safety profile of Se-AgNPs remains uncertain. It is also important to note that one of the included studies is subject to an expression of concern regarding the reliability of its data obtained by energy-dispersive X-ray spectroscopy (EDX) and scanning electron microscopy coupled with energy-dispersive X-ray spectroscopy (SEM/EDX) [7]. As this represents a single dataset, it does not affect the overall consistency of the trends observed across the remaining literature [7].

 Despite promising antimicrobial activity and reduced cytotoxicity, several translational and regulatory barriers still limit the clinical application of Se-AgNP-based biomaterials [63, 64]. Key challenges include the lack of long-term in vivo evidence on biodistribution, systemic accumulation, chronic toxicity, and immunological and environmental effects following NPs exposure [63, 64]. In parallel, issues related to large-scale synthesis reproducibility, colloidal stability, sterilization methods, storage conditions, and manufacturing scalability remain insufficiently addressed [63, 64]. From a regulatory standpoint, the absence of standardized nanotoxicological frameworks further complicates clinical translation, as regulatory approval requires comprehensive characterization of NPs composition, degradation behavior, ion release kinetics, pharmacokinetics, and safety profiles [63, 64]. However, most available studies provide limited information on these parameters, particularly regarding batch consistency and long-term biological stability, thereby constraining their translational readiness.

Despite variability in NP size, synthesis routes, and experimental protocols, Se-AgNPs consistently demonstrate superior antimicrobial performance compared to isolated NPs. Future investigations should prioritize standardized experimental designs, including defined concentrations, delivery systems, and integrated biological evaluation frameworks encompassing antimicrobial efficacy, cytocompatibility, and genetic safety. In addition, the use of relevant mammalian cell models and well-controlled in vivo systems will be essential to generate robust evidence on safety and efficacy, thereby strengthening the translational potential of Se-AgNPs for advanced biomaterials in infection control and tissue regeneration.

## Conclusion

Based on the currently available evidence, Se-AgNPs showed a synergistic enhancement of antimicrobial and antibiofilm activity, with a potential reduction in cytotoxic effects. Nevertheless, the small number of studies limits the strength of the conclusions, underscoring the need for rigorous, standardized investigations to strengthen or refute these findings.

## Supplementary Information

Below is the link to the electronic supplementary material.

Supplementary Material 1 (DOCX 22.2 KB)

## Data Availability

No datasets were generated or analysed during the current study.
