# Supplementary material for: Addition of Selenium to Silver Nanoparticles: An Approach to Enhance Their Antimicrobial and Biological Properties – Systematic Review
Source: Biol Trace Elem Res. 2026 Jun 25;204(9):7226–42. doi: 10.1007/s12011-026-05184-5 (PMC13437565; doi:10.1007/s12011-026-05184-5)
Supplement: Supplementary file 1 [file 12011_2026_5184_MOESM1_ESM.docx]

**Supplementary Table 1.** Search strategy and results.

| **Databases** | **Search strategy** | **Filter** | **Results** |
| --- | --- | --- | --- |
| **PubMed** | (("Selenium" OR "SeNPs") AND ("Silver" OR "Silver nanoparticles" OR "Ag" OR "AgNPs") AND ("Antimicrobial" OR "Microbial" OR "Antibiofilm" OR "Antibacterial" OR "Antifungal" OR "Biological" OR "Biocompatibility" OR "Cytotoxicity" OR "Genotoxicity" OR "Mutagenicity" OR "Cell Toxicity")) | None | 211 |
| **Science Direct** | (("Selenium") AND ("Silver nanoparticles") AND ("Microbial" OR "Biological" OR "Biocompatibility" OR "Cytotoxicity" OR "Genotoxicity" OR "Mutagenicity" OR "Cell Toxicity")) | Research articles | 543 |
| **Embase** | (("Selenium" OR "SeNPs") AND ("Silver" OR "Silver nanoparticles" OR "Ag" OR "AgNPs") AND ("Antimicrobial" OR "Microbial" OR "Antibiofilm" OR "Antibacterial" OR "Antifungal" OR "Biological" OR "Biocompatibility" OR "Cytotoxicity" OR "Genotoxicity" OR "Mutagenicity" OR "Cell Toxicity")) | ('animal cell'/de OR 'animal experiment'/de OR 'animal model'/de OR 'animal tissue'/de OR 'biological model'/de OR 'cell culture'/de OR 'in vitro study'/de OR 'methodology'/de) | 276 |
| **Scopus** | (("Selenium" OR "SeNPs") AND ("Silver" OR "Silver nanoparticles" OR "Ag" OR "AgNPs") AND ("Antimicrobial" OR "Microbial" OR "Antibiofilm" OR "Antibacterial" OR "Antifungal" OR "Biological" OR "Biocompatibility" OR "Cytotoxicity" OR "Genotoxicity" OR "Mutagenicity" OR "Cell Toxicity")) | Research articles | 634 |
| **Lilacs** | (("Selenium" OR "SeNPs") AND ("Silver" OR "Silver nanoparticles" OR "Ag" OR "AgNPs") AND ("Antimicrobial" OR "Microbial" OR "Antibiofilm" OR "Antibacterial" OR "Antifungal" OR "Biological" OR "Biocompatibility" OR "Cytotoxicity" OR "Genotoxicity" OR "Mutagenicity" OR "Cell Toxicity")) | None | 5 |
| **Google Scholar** | (("Selenium" OR "SeNPs") AND ("Silver" OR "Silver nanoparticles" OR "Ag" OR "AgNPs") AND ("Antimicrobial" OR "Microbial" OR "Antibiofilm" OR "Antibacterial" OR "Antifungal" OR "Biological" OR "Biocompatibility" OR "Cytotoxicity" OR "Genotoxicity" OR "Mutagenicity" OR "Cell Toxicity")) | None | 0 |
| **ProQuest** | (("Selenium" OR "SeNPs") AND ("Silver" OR "Silver nanoparticles" OR "Ag" OR "AgNPs") AND ("Antimicrobial" OR "Microbial" OR "Antibiofilm" OR "Antibacterial" OR "Antifungal" OR "Biological" OR "Biocompatibility" OR "Cytotoxicity" OR "Genotoxicity" OR "Mutagenicity" OR "Cell Toxicity")) | None | 0 |

**Supplementary Table 2.** Excluded studies and reasons for exclusion.

| **Author and year** | **Reasons for exclusion** |
| --- | --- |
| Adam et al., 2015 | 1 |
| Aksakal et al., 2018 | 1, 2 |
| Aksakal et al., 2023 | 1, 2 |
| Ansar et al., 2017 | 3 |
| Bankier et al., 2019 | 2 |
| Biswas et al., 2018 | 1, 2 |
| Chudobova et al., 2014 | 2 |
| Dias et al., 2024 | 2 |
| Elattar et al., 2024 | 2 |
| Eslamieh-ei et al., 2023 | 2 |
| Hameed et al., 2025 | 2 |
| Jamróz et al., 2023 | 1, 2 |
| Krishnaraj et al., 2022 | 1, 2 |
| Krishnaraj et al., 2024 | 1 |
| Kuzmenko et al., 2018 | 2, 4 |
| Liu et al., 2021 | 2 |
| Lotfali et al., 2021 | 2 |
| Malinga et al., 2021 | 2, 4 |
| Menazea et al., 2022 | 2 |
| Mirzapoor et al., 2023 | 2 |
| Mittal et al., 2014 | 4 |
| Mittal et al., 2021 | 2, 4 |
| More et al., 2015 | 2, 4 |
| Mosa et al., 2022 | 2 |
| Mostafa et al., 2023 | 2 |
| Naganuma et al., 1983 | 1, 2 |
| Nie et al., 2020 | 1 |
| Olawale et al., 2021 | 2, 4 |
| Perfileva et al., 2018 | 1 |
| Reddy et al., 2023 | 2 |
| Ren et al., 2024 | 2 |
| Say et al., 2019 | 1, 2 |
| Say et al., 2022 | 1, 2 |
| Shaaban et al., 2018 | 2 |
| ShekoftehNarm et al., 2025 | 1, 2 |
| Singh et al., 2014 | 2 |
| Singh et al., 2015 | 2 |
| Staats et al., 2022 | 2 |
| Subramanian et al., 2022 | 2 |
| Surya et al., 2024 | 2 |
| Tsivileva et al., 2021 | 2 |
| Verma et al., 2018 | 2, 4 |
| Vrandečić et al., 2020 | 1, 2 |

**Legend:** 1. Studies involving the association of Ag and Se with other compounds; 2. Studies that did not compare the association between Ag and Se; 3. Studies evaluating other biological outcomes (e.g., inflammation); and . Studies without microbiological testing.
